# Characterization of Protein Radicals in Arabidopsis

**DOI:** 10.3389/fphys.2019.00958

**Published:** 2019-08-13

**Authors:** Aditya Kumar, Ankush Prasad, Michaela Sedlářová, Pavel Pospíšil

**Affiliations:** ^1^Department of Biophysics, Centre of the Region Haná for Biotechnological and Agricultural Research, Faculty of Science, Palacký University Olomouc, Olomouc, Czechia; ^2^Department of Botany, Faculty of Science, Palacký University Olomouc, Olomouc, Czechia

**Keywords:** aggregate, fragment, photosystem II, protein, reactive oxygen species, singlet oxygen, hydroxyl radical, protein radical

## Abstract

Oxidative modification of proteins in photosystem II (PSII) exposed to high light has been studied for a few decades, but the characterization of protein radicals formed by protein oxidation is largely unknown. Protein oxidation is induced by the direct reaction of proteins with reactive oxygen species known to form highly reactive protein radicals comprising carbon-centered (alkyl) and oxygen-centered (peroxyl and alkoxyl) radicals. In this study, protein radicals were monitored in Arabidopsis exposed to high light by immuno-spin trapping technique based on the detection of 5,5-dimethyl-1-pyrroline N-oxide (DMPO) nitrone adducts using the anti-DMPO antibody. Protein radicals were imaged in Arabidopsis leaves and chloroplasts by confocal laser scanning microscopy using fluorescein conjugated with the anti-DMPO antibody. Characterization of protein radicals by standard blotting techniques using PSII protein specific antibodies shows that protein radicals are formed on D1, D2, CP43, CP47, and Lhcb3 proteins. Protein oxidation reflected by the appearance/disappearance of the protein bands reveals that formation of protein radicals was associated with protein fragmentation (cleavage of the D1 peptide bonds) and aggregation (cross-linking with another PSII subunits). Characterization of protein radical formation is important for better understating of the mechanism of oxidative modification of PSII proteins under high light.

## Introduction

Plants are prone to various environmental stresses throughout their life cycle as high light, UV irradiation, heat, cold, drought and salinity (Murata et al., 2007; Choudhury et al., 2017). Plant responses to various types of stress factor by the formation of reactive oxygen species (ROS) are known to play a crucial role in the retrograde signaling and oxidative damage (Schmitt et al., 2014; Laloi and Havaux, 2015; Dietz et al., 2016; Mittler, 2017). Under high light, ROS are produced when absorption of light energy by chlorophylls surpasses the capacity for its use in the photosynthetic reactions. It is evidenced that photosystem II (PSII) produces various types of ROS at different sites (Pospíšil, 2012; Fischer et al., 2013; Telfer, 2014). Singlet oxygen (^1^O_2_) is formed by energy transfer from triplet chlorophyll to molecular oxygen at the site of coordination of weakly-coupled or uncoupled chlorophylls in the PSII antenna (CP43, CP47, and Lhcb proteins) and PSII reaction center (D1, D2) proteins. Hydroxyl radical (HO^∙^) is formed by metal-mediated reduction of hydrogen peroxide at the site of metal coordination to PSII reaction center proteins (Pospíšil, 2014). It is well accepted that both ^1^O_2_ and HO^∙^ oxidize PSII proteins and thus alter subsequently the structure of the PSII proteins. The oxidation of protein by ROS forms carbon-centered (alkyl) and oxygen-centered (peroxyl and alkoxyl) protein radicals (Davies, 2016). In spite of the fact that oxidative modification of PSII proteins has been intensively studied in past decades, the formation of protein radicals by oxidation of PSII proteins was not explored in detail. It was predominantly due to a limitation in the detection of protein radicals caused by high reactivity of protein radicals toward other proteins and short lifetime of protein radicals (Mattila et al., 2015).

It has been previously established that immuno-spin trapping technique which combines the specificity and sensitivity of spin trapping with various immunoassays is suitable method for the detection of protein radicals in animal cells (Gomez-Mejiba et al., 2014). In this technique, the reaction of spin trap 5,5-dimethyl-1-pyrroline N-oxide (DMPO) with an organic (proteins, lipids, and nucleic acids) radical forms a stable nitrone adduct (DMPO-P adduct), which is identified by an anti-DMPO antibody raised against nitrone moiety of the adduct (Mason, 2016). Recently, protein radicals have been visualized for the first time in Arabidopsis plants using the immuno-spin trapping technique (Kumar et al., 2019). The authors showed by confocal laser scanning microscopy using fluorescein conjugated with anti-DMPO antibody that protein radicals are formed predominantly in the chloroplasts located at the periphery of the cells and distributed uniformly throughout the grana stack. To understand the mechanism of oxidative reactions, it is very important to analyze which PSII proteins are oxidized by ROS. Using tandem mass spectroscopy, natively oxidized amino acid residues in PSII membranes isolated from field-grown spinach showed that oxidized amino acids are localized in the vicinity of the Mn_4_O_5_Ca cluster, PheoD1 (D1 residues 130E, 133L and 135F) and Q_A_ (D1 residues 214Q, 239F, and 242E) (Frankel et al., 2012, 2013). Detail analysis of amino acid oxidized in spinach PSII membranes under high light showed that amino acids located in the close proximity to the metal coordinated to D1 and D2 proteins are oxidized (Kale et al., 2017). The authors showed that D1:332H coordinated to the Mn_4_O_5_Ca cluster and D2:244Y coordinated to the non-heme iron via bicarbonate are oxidized by HO^∙^. Similarly, identification of oxidized residues on the luminal side of PSII in the cyanobacterium *Synechocystis* sp. PCC 6803 showed that D1:332H is oxidized under high light (Weisz et al., 2017). The authors proposed that oxidized amino acid forms wall of the water channel through which ROS formed at the Mn_4_O_5_Ca cluster are driven away from the catalytic center to the lumen.

In this study, we used immuno-spin trapping to monitor the formation of protein radicals in Arabidopsis. Standard blotting techniques using various antibodies to PSII proteins provided a complete characterization of protein radical formed on various PSII proteins involved in oxidative processes.

## Materials and Methods

### Plant Material, Leaf, Chloroplast and Thylakoid Membrane Isolation

Seeds of wild-type Arabidopsis (*Arabidopsis thaliana*, cv. Columbia-0; WT) obtained from the Nottingham Arabidopsis Stock Center (NASC), University of Nottingham (Loughborough, United Kingdom) were soaked in distilled water and then potted in growing pots with a peat substrate (Klasmann, Potgrond H). Plants were grown in a growing chamber (Photon Systems Instruments, Drásov, Czechia) under controlled conditions with a light intensity of 100 μmol photons m^–2^ s^–1^, photoperiod of 8/16 h and temperature of 25°C (unless specified otherwise) with a relative air humidity of 60%. Arabidopsis plants (5 or 6 weeks old) were exposed to high light stress (1500 μmol photons m^–2^ s^–1^) at a low air temperature of 8°C for 13 h using AlgaeTron AG 230 (Photon Systems Instruments, Drásov 470, Czechia). Leaves were cut into 3–5 mm small square pieces using surgical knife. Intact chloroplasts were prepared using Percoll gradient centrifugation as described by Seigneurin-Berny et al. (2008). Both sliced leaf pieces and isolated chloroplasts were used immediately for confocal measurements. Thylakoid membranes were prepared according to Casazza et al. (2001) and stored at −80°C in the dark until use.

### *In vitro* Detection of Reactive Oxygen Species by Electron Paramagnetic Resonance Spectroscopy

Detection of ^1^O_2_ and HO^∙^ was performed by electron paramagnetic resonance (EPR) spectroscopy using an EPR spectrometer (MiniScope MS400, Magnettech GmbH, Berlin, Germany). For ^1^O_2_ detection, a hydrophilic diamagnetic compound TMPD (2, 2, 6, 6-tetramethyl-4-piperidone) was used (Moan and Wold, 1979) while for HO^∙^, POBN (4-pyridyl-1-oxide-N-tert-butylnitrone)/ethanol system was used (Pou et al., 1994). For validation of spin trap-compounds, chemically generated ^1^O_2_ and HO^∙^ were utilized (Supplementary Datasheet S1) and data simulation were performed (for details, refer to Supplementary Datasheet S2). EPR spectra were collected using following conditions: microwave power (10 mW), modulation amplitude (0.1 mT), modulation frequency (100 kHz), sweep width (8 mT) and sweep time (60 s). EPR signal intensity was evaluated from the relative height of the central peak of TEMPONE EPR spectrum or central doublet peak of POBN-CH(CH_3_)OH adduct EPR spectrum. Simulation of EPR spectra was performed by WinSim software (National Institute of Environmental Health Sciences, Research Triangle Park, NC, United States).

### *In vivo* Imaging of Reactive Oxygen Species by Confocal Laser Scanning Microscopy

Formation of ^1^O_2_ and HO^∙^ in leaves was visualized by confocal laser scanning microscopy (Fluorview 1000 unit attached to IX80 microscope; Olympus Czech Group, Prague, Czechia) (Kumar et al., 2018; Prasad et al., 2018). Leaf pieces were incubated either in dark or red light (1000 μmol photons m^–2^ s^–1^) at room temperature with 50 μM Singlet Oxygen Sensor Green (SOSG) for the detection of ^1^O_2_ and 5 μM Hydroxy Phenyl Fluorescein (HPF) for the detection of HO^∙^ in the presence of HEPES buffer (pH 7.5). Singlet oxygen imaging was based on its interaction with SOSG forming SOSG endoperoxide (SOSG-EP) while HO^∙^ imaging was based on its interaction with HPF forming HPF-ox; both products provide bright green fluorescence. The excitation of SOSG and HPF was achieved by 488 nm line of an argon laser and emission was detected at 505–525 nm using BA505-525 filter (Olympus).

### *In vivo* Imaging of Protein Radicals by Confocal Laser Scanning Microscopy

Formation of protein radicals in leaves and chloroplasts was visualized by confocal laser scanning microscopy (Kumar et al., 2019). Sliced leaf pieces and chloroplasts were incubated either in dark or red light (1000 μmol photons m^–2^ s^–1^) for 30 min at room temperature, in the presence of MES-NaOH buffer (40 mM, pH 6.5), spin trap DMPO (50 mM), anti-DMPO antibody (5 μg ml^–1^) conjugated with fluorescein isothiocyanate (FITC) and Triton X-100 (0.001%). Triton X-100 was used in order to increase the penetration of spin trap and antibody through the cell wall and membrane. Protein radicals were imaged based on their interaction with DMPO forming DMPO-protein radical adduct known to disproportionate to stable DMPO-nitrone adduct which is recognized by anti-DMPO antibody conjugated with FITC. Anti-DMPO is claimed by the manufacturer (Abcam, Cambridge, United Kingdom) for its non-cross reactivity with non-adducted proteins or DNA; however, it can recognize free DMPO and thus should be taken into account. FITC fluorescence in leaf and chloroplast was visualized by confocal laser scanning microscopy. The excitation of FITC was achieved by 488 nm line of an argon laser and emission was detected at 505–525 nm using BA505–525 filter (Olympus).

### SDS-PAGE and Immuno-Spin Trapping

To visualize the protein degradation/loss during high light illumination, thylakoid membranes (5 μg Chl) isolated from long term high light exposed plants at low air temperature (13 h, 1500 μmol photons m^–2^ s^–1^, 8°C) was incubated either in dark or in high white light (1500 μmol photons m^–2^ s^–1^) for 10, 20, and 30 min in the presence of DMPO (50 mM) spin trap at room temperature. After dark/light incubation, protein extraction using DTT (dithiothreitol) protein extraction buffer followed by heating at 70°C in the dry bath for 15 min and centrifugation at 16,000 × *g* for 5 min at 4°C was performed. The supernatant was loaded into wells and SDS-PAGE was completed using a Tris-Tricine SDS-PAGE protocol described by Schagger (2006) using Mini-PROTEAN Tetra vertical electrophoresis cell (Bio-Rad, CA, United States). Proteins resolved in SDS gels were either stained with Coomassie Brilliant Blue (CBB) R-250 in a methanol/acetic acid solution followed by de-staining to remove the high blue background (Brunelle and Green, 2014) or transferred to a nitrocellulose (NC) membrane using a semi-dry blotter (*Trans-*Blot SD, Semi-dry transfer cell, Bio-Rad, United States). To detect protein radicals formed on the PSII proteins (reaction center proteins and/or antenna complex proteins) or on their aggregate/cleaved peptide, blocking step of NC membrane was done with 5% BSA prepared in phosphate buffered saline-tween 20 (PBST; pH- 7.4) at 4°C overnight to prevents antibodies from binding to the membrane non-specifically. All successive steps were performed on a shaker at room temperature. After blocking, the NC membrane was incubated with rabbit polyclonal anti-DMPO nitrone adduct primary antibody (1:5000, Abcam) raised against DMPO followed by 10 min, three to five washes with PBST and 1 h incubation with horseradish peroxidase (HRP) conjugated goat anti-rabbit secondary antibody (1:10000, Bio-Rad) and protein bands were visualized using luminol as a chemiluminescent probe and images were captured by AI600 (Amersham Imager 600, GE Health Care Europe GmbH, Freiburg, Germany). For the identification of the origin of protein bands, we used specific antibodies from Agrisera raised against PSII proteins. Antibodies used are anti-D1, anti-C-terminal, anti-D-*de* loop, anti-cyt *b*_559_ α-subunit, anti-D2, anti-CP43, anti-CP47 and anti-Lhcb3. Size of protein bands was determined using a standard protein ladder (PageRuler^TM^ Pre-stained Protein Ladder, 10 to 180 kDa, Thermo Fisher Scientific, Lithuania). Densitometry of western blots was performed with ImageJ (National Institute of Mental Health, Bethesda, MD, United States) and the quantification of protein band intensities was shown as peaks in densitogram. The area under the peak was evaluated to determine the increase or decrease of protein band intensities in the SDS-gel and blots probed with different PSII protein antibodies.

## Results

### Reactive Oxygen Species Formation Under High Light Stress in Arabidopsis

**FIGURE 1:**
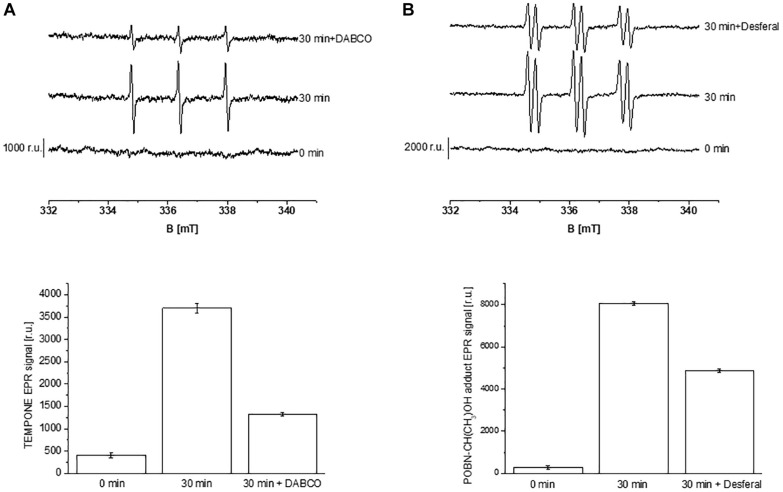
Detection of singlet oxygen **(A)** and hydroxyl radical **(B)** in Arabidopsis thylakoid membranes by electron paramagnetic resonance spectroscopy. **(A)** Thylakoid membranes (50 μg Chl ml^–1^) in 50 mM TMPD were kept in the dark (0 min) and illuminated with high white light (1500 μmol photons m^–2^ s^–1^, 30 min) in the absence and presence of 25 mM DABCO. **(B)** Thylakoid membranes (100 μg Chl ml^–1^) in 50 mM POBN containing 170 mM ethanol were kept in the dark (0 min); high white light (1500 μmol photons m^–2^ s^–1^, 30 min) in the absence and presence of 5 mM desferal. The lower panels show the mean value and standard deviation of EPR signal intensity (*n* = 3).

**FIGURE 2:**
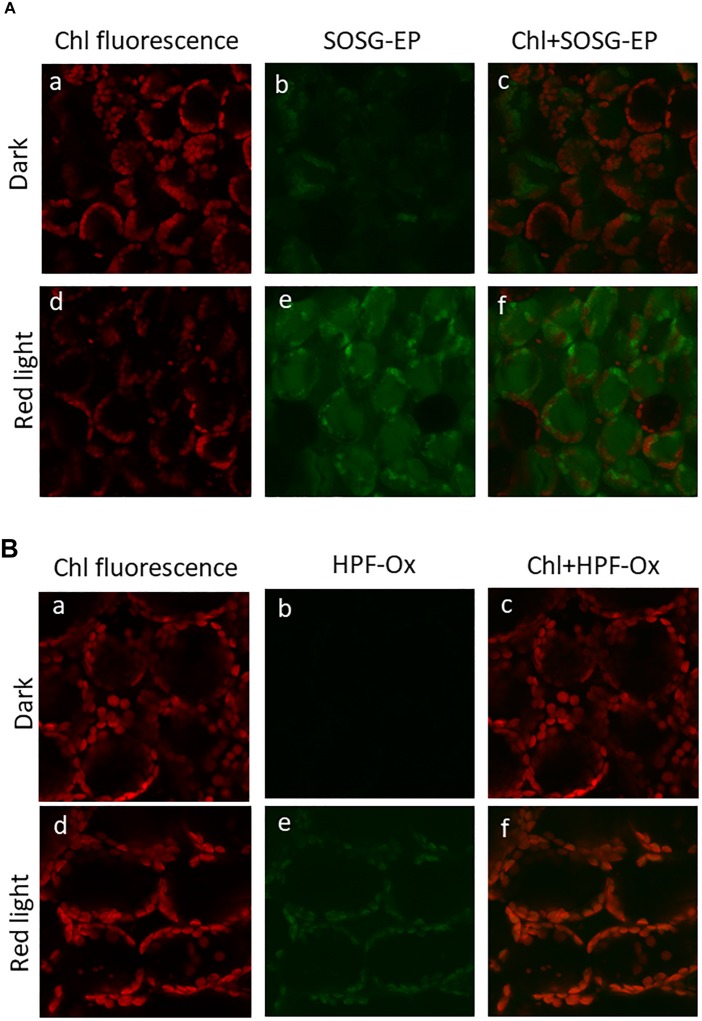
Imaging of singlet oxygen **(A)** and hydroxyl radical **(B)** in Arabidopsis leaves monitored by laser confocal scanning microscopy. **(A)** Singlet oxygen imaging was achieved by infiltration with 50 μM SOSG in dark (upper panel) or exposed to high red light (1000 μmol photons m^–2^ s^–1^) (lower panel) for 30 min. From left to right is chlorophyll fluorescence channel **(a,d)**; SOSG-EP fluorescence channel **(b,e)** and combination of chlorophyll fluorescence + SOSG-EP fluorescence channel **(c,f)**. **(B)** Hydroxyl radical imaging was achieved by infiltration with 5 μM HPF in dark (upper panel) or exposed to high red light (lower panel) for 30 min. From left to right is chlorophyll fluorescence channel **(a,d)**; HPF-ox fluorescence channel **(b,e)** and combination of chlorophyll fluorescence + HPF-ox fluorescence channel **(c,f)**.

Formation of ROS (^1^O_2_ and HO^∙^) in the thylakoid membranes exposed to high white light was measured by EPR spectroscopy using TMPD spin probe and POBN/ethanol spin trap system, respectively. No EPR signal was observed in dark (Figures 1A,B, trace 0 min), whereas illumination with continuous white light for 30 min resulted in the formation of the 2,2,6,6-tetramethyl-4-piperidone-1-oxyl (TEMPONE) EPR and α-hydroxyethyl radical adduct of POBN [POBN-CH(CH_3_)OH adduct] EPR signals (Figures 1A,B, trace 30 min). Significant suppression in ^1^O_2_ and HO^∙^ were observed in the presence of ^1^O_2_ quencher, diazabicyclo [2.2.2] octane (DABCO) and iron chelator, deferoxamine (desferal) known to prevent HO^∙^ formation. The formation of ^1^O_2_ and HO^∙^ in Arabidopsis leaves exposed to high white light was validated by confocal laser scanning microscopy using SOSG and HPF fluorescent probes, respectively. Negligible fluorescence was observed in non-illuminated leaves (Figures 2Ab,Bb), whereas illumination with continuous high red light for 30 min resulted in the formation of SOSG-EP and HPF-ox fluorescence (Figures 2Ae,Be). These results indicate that exposure of Arabidopsis to high light results in the formation of ^1^O_2_ and HO^∙^.

### Imaging of Protein Radicals by Confocal Laser Scanning Microscopy

**FIGURE 3:**
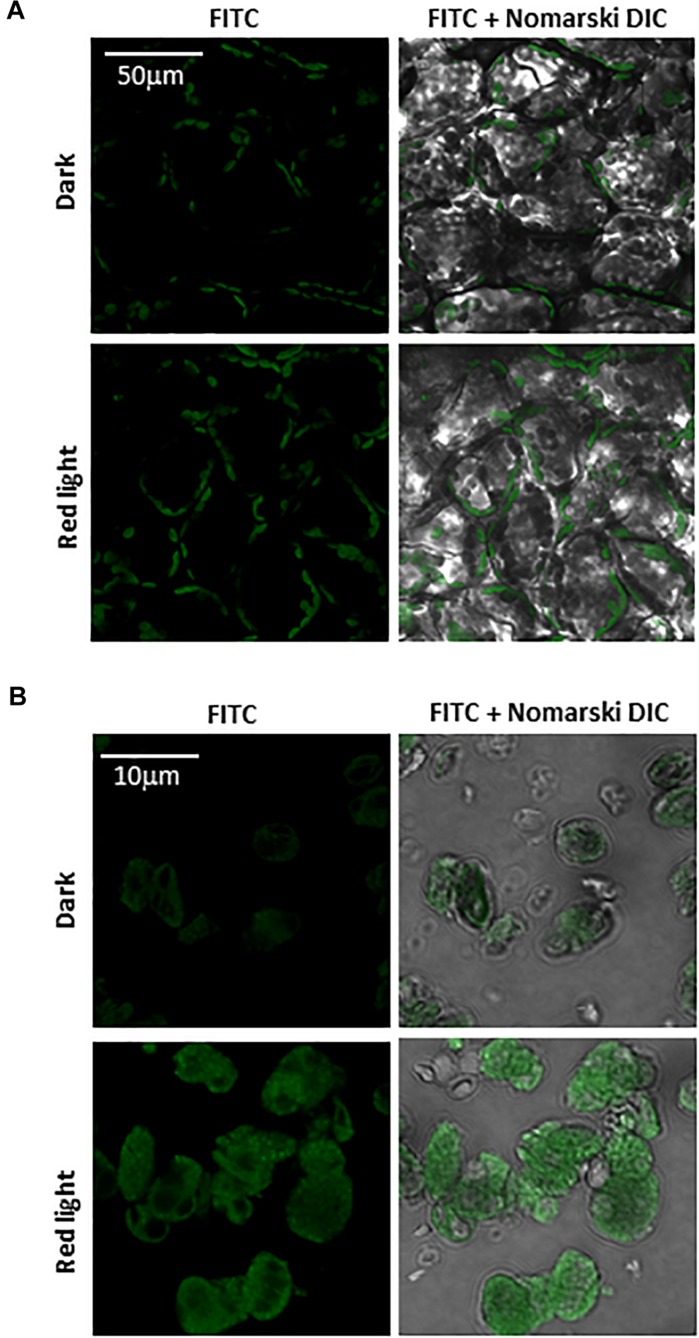
Protein radical imaging in Arabidopsis leaves **(A)** and chloroplasts **(B)** monitored by laser confocal scanning microscopy. From left to right: FITC fluorescence channel, the combination of FITC fluorescence and Nomarski DIC channels are shown. Prior to FITC fluorescence measurement, Arabidopsis leaves and chloroplasts were kept in the dark or illuminated with red light (1000 μmol photons m^–2^ s^–1^) for 30 min in the presence of 50 mM DMPO and 5 μg ml^–1^ anti-DMPO nitrone adduct antibody conjugated with FITC.

Imaging of protein radicals in Arabidopsis was performed by confocal laser scanning microscopy using the immuno-spin trapping technique. Figures 3A,B show imaging of protein radicals in Arabidopsis leaves previously exposed to high white light at low temperature (1500 μmol photons m^–2^ s^–1^, 13 h, 8°C) and chloroplasts isolated from high light exposed Arabidopsis leaves, respectively. In immuno-spin trapping technique, short-lived protein radicals (*t*_1__/__2_ ∼ μs) are trapped by spin trap DMPO to form a more stable DMPO-protein radical adduct (*t*_1__/__2_ ∼ min). The DMPO-protein radical adduct is reduced to diamagnetic DMPO nitrone adduct in the reducing environment which is detected by anti-DMPO antibody conjugated with FITC and imaged by confocal laser scanning microscopy. FITC fluorescence was measured in Arabidopsis leaves and chloroplasts treated with DMPO spin trap and anti-DMPO antibody conjugated with FITC either in the dark or high red light (Figure 3). Figure 3 shows the FITC fluorescence channel (left panel) and the combination of FITC fluorescence and Nomarski DIC channels (right panel). Arabidopsis leaves and chloroplasts treated in dark showed low FITC fluorescence, whereas leaves and chloroplasts exposed to high red light showed a significant increase in FITC fluorescence due to the formation of protein radicals under high red light. It is noticeable that protein radicals are formed in chloroplasts located at the periphery of mesophyll cells.

### Separation of PSII Proteins by SDS-PAGE

**FIGURE 4:**
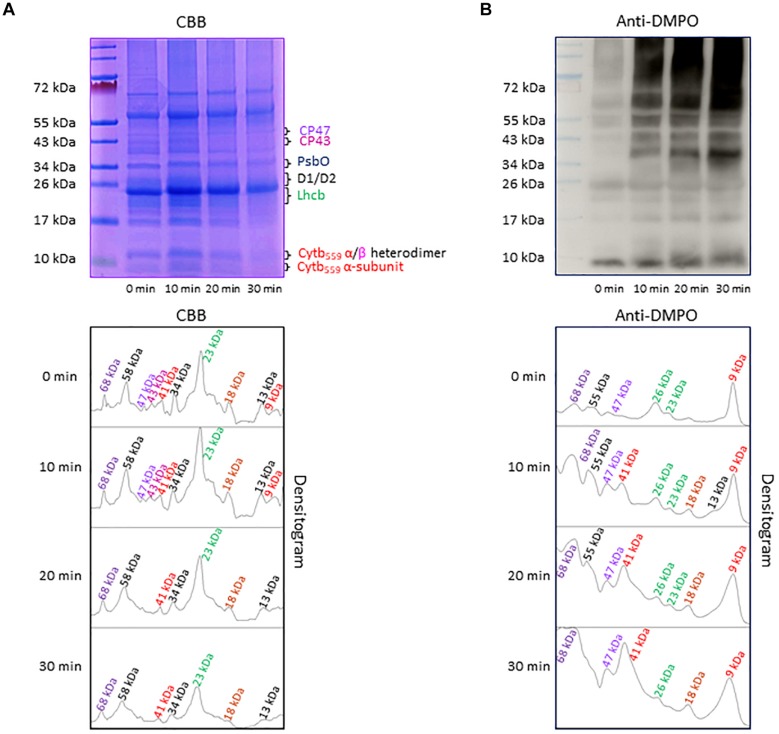
**(A)** Separation of PSII proteins by SDS-PAGE. Pre-stained standard protein ladder (*lane 1*), thylakoid membranes incubated in dark (*lane 2*), thylakoid membranes incubated in high white light for 10, 20, and 30 min (*lane 3, lane 4*, and *lane 5*, respectively). The identity of the proteins is as per their mass. **(B)** Protein radical detection in thylakoid membranes using immuno-spin trapping technique. Pre-stained standard protein ladder (*lane 1*), thylakoid membranes incubated in dark in presence of DMPO (*lane 2*), thylakoid membranes exposed to high light in the presence of DMPO for 10, 20 and 30 min (*lane 3, lane 4*, and *lane 5*, respectively). Analysis of DMPO-protein nitrone adducts in thylakoid membranes incubated with DMPO under high white light for different time points. Indicated are the protein radicals formed on proteins, protein fragments and protein aggregates. In panels **(A,B)**, the densitograms (lower panels) show the number and density of the protein bands in respective lanes. Peaks in the densitogram are labeled with an apparent size of the protein determined using a standard protein ladder protein marker. Color code is used to mark the different proteins in densitogram.

Separation of PSII proteins was performed using SDS-PAGE of thylakoid membranes isolated from high light exposed Arabidopsis leaves and visualization of protein bands was achieved by CBB staining (Figure 4A). According to band size based on the use of a standard protein ladder, it was found that protein bands appeared at 9 kDa (α-subunit of cyt *b*_559_), 13 kDa (α- and β-subunit heterodimer of cyt *b*_559_), 23 kDa (Lhcb), 30–32 kDa (D1/D2), 33 kDa (PsbO), 43 kDa (CP43) and 47 kDa (CP47). Apart from these bands, low-molecular weight bands at 18 kDa and high-molecular weight bands at 41, 58, and 68 kDa were observed. The protein band at 18 kDa is assigned to fragments formed by the cleavage of PSII proteins, whereas the protein bands at 41 and 68 kDa represent aggregates formed by the cross-linking of PSII proteins. The protein band at 58 kDa might be assigned to ATPase and/or PsaA. The band density increased after exposure of the thylakoid membranes to high red light for 10 min and subsequently decreased. These results reveal the formation of protein fragments and aggregates after exposure of Arabidopsis plant to high light and degradation of proteins, their fragments and aggregates due to continuous oxidation.

### Formation of Protein Radical Detected by Immuno-Spin Trapping

To explore the formation of protein radicals on the PSII proteins, immunoblot analysis of the thylakoid membranes isolated from high white light exposed plants was accomplished (Figure 4B). DMPO protein nitrone adducts formed on different proteins separated by SDS-PAGE were transferred to the NC membrane, identified by anti-DMPO nitrone adduct antibody and visualized by luminol as a chemiluminescent probe using HRP conjugated secondary antibody. In dark, weak protein bands at 9, 23, 26, 47, 55, and 68 kDa were observed which can be due to binding of DMPO to some proteins other than protein radicals as reported recently (Munoz et al., 2019). After exposure to high white light, the intensity of 9, 47, 55, and 68 kDa protein bands increased and two new protein bands appeared at 18 and 41 kDa. Quantification of protein bands in each lane of the blot by densitogram showed four times increase in 41 and 68 kDa protein band density and two times increase in 18 kDa protein band density. These results reveal the formation of protein radicals on protein aggregates and fragments.

### Characterization of PSII Reaction Center Proteins by Western-Blotting

**FIGURE 5:**
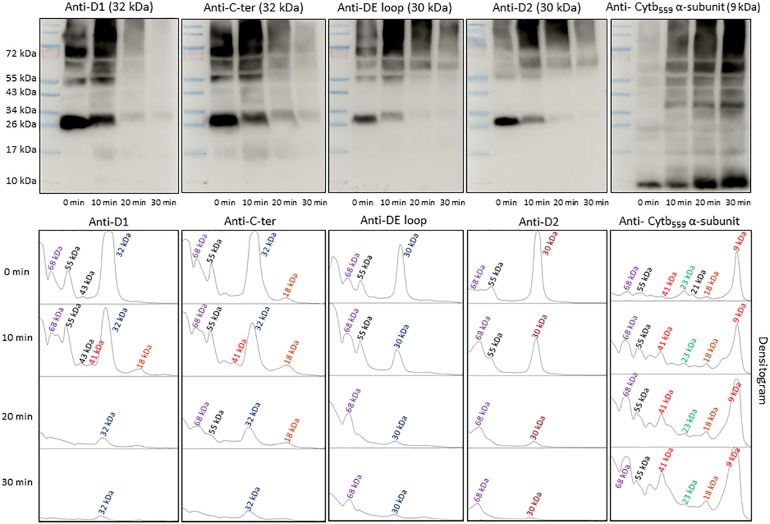
Identification of PSII reaction center proteins by Western-Blotting. Pre-stained standard protein ladder (*lane 1*), thylakoid membranes incubated in dark (*lane 2*), thylakoid membranes incubated in high white light for 10, 20, and 30 min (*lane 3, lane 4*, and *lane 5*, respectively). Immunoblot of dark-adapted and illuminated thylakoid membranes incubated with DMPO and identified with an anti-D1, anti-C-terminal, anti-D-*de* loop, anti-D2 and anti-cytb_559_ α-subunit antibodies (*left to right*). The densitograms (lower panels) represent the number and density of the protein bands in respective lanes.

To identify the origin of protein bands in anti-DMPO blot, the NC membranes were probed with different PSII protein antibodies (Figure 5). To detect the protein bands originated from the D1, D2 and α-subunit of cyt *b*_559_ protein, antibodies raised against the D1 protein (anti-D1 antibody), the C-terminal of the D1 protein (anti-C-terminal antibody), the D-*de* loop of the D1 protein (anti-D-*de* loop antibody), the D2 protein (anti-D2 antibody) and the α-subunit of cyt *b*_559_ protein (anti-cyt *b*_559_ α-subunit antibody) were used. When the blot was probed with an anti-D1 antibody, anti-C-terminal antibody, anti-D-*de* loop antibody and anti-D2 antibody, protein bands with an apparent molecular weight of 32 and 30 kDa were detected. In addition, one protein band below 32 kDa D1 protein band with apparent molecular weight of 18 kDa and two protein bands above 32 kDa D1 protein band with apparent molecular weights of 55 and 68 kDa were observed. When the blot was probed with an anti-cyt *b*_559_ α-subunit antibody, 9 and 41 kDa protein bands were observed. After exposure to high red light, a significant decrease in the protein band densities of D1 and D2 proteins was observed. Interestingly, the band densities of 9 and 41 kDa proteins increased under high red light. Quantification of protein bands in each lane of anti-D1 antibody, anti-C-terminal antibody, anti-D-*de* loop antibody, anti-D2 antibody and anti-cyt *b*_559_ α-subunit antibody blot by densitograms reveals a several fold decrease in D1, C-terminal, D-*de* loop and D2 protein band density and three times increase in α-subunit of cyt *b*_559_ protein band density. Comparison of these blots with anti-DMPO blot of the same samples performed in parallel suggests the contribution of D1, C-terminal, D-*de* loop, D2 and α-subunit of cyt *b*_559_ proteins in 55 and 68 kDa aggregate formation, and contribution of D1, C-terminal and α-subunit of cyt *b*_559_ protein in 41 and 18 kDa aggregate formation. These results provide clear evidence on the involvement of D1, C-terminal, D-*de*-loop and α-subunit of cyt *b*_559_ proteins in the aggregation and protein radical formation on the aggregates.

### Characterization of PSII Antenna Complex Protein Radicals by Western-Blotting

**FIGURE 6:**
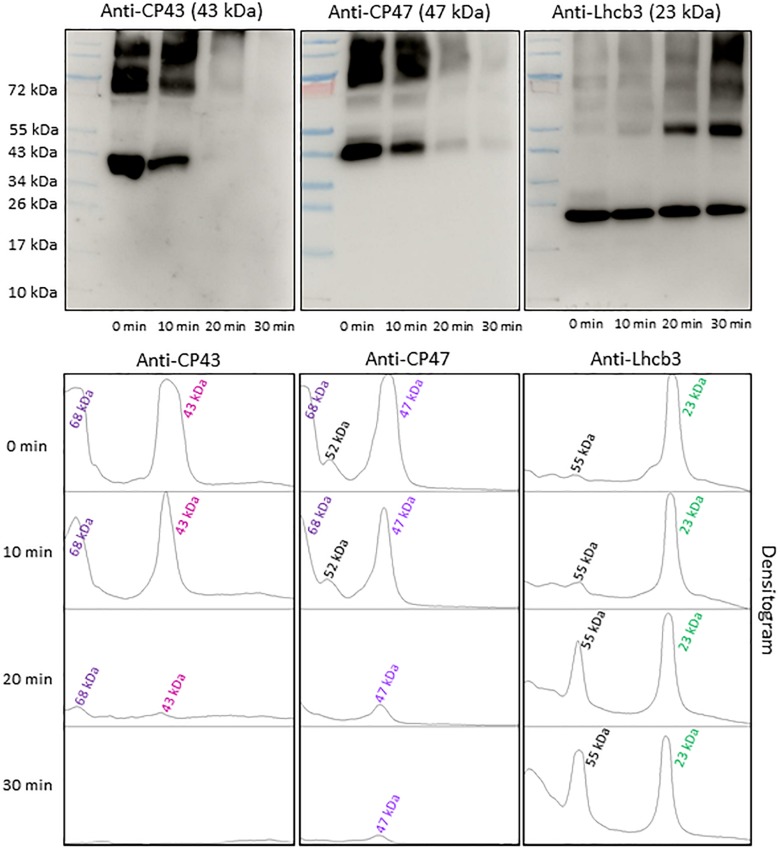
Identification of PSII antenna complex proteins by Western-Blotting. Pre-stained standard protein ladder (*lane 1*), thylakoid membranes incubated in dark (*lane 2*), thylakoid membranes incubated in high white light for 10, 20, and 30 min (*lane 3, lane 4*, and *lane 5* respectively). Immunoblot of dark-adapted and illuminated thylakoid membranes incubated with DMPO and identified with an anti-CP43, anti-CP47 and anti-Lhcb3 antibodies (*left to right*). The densitograms (lower panels) show the number and density of the protein bands in respective lanes. The number of peaks in densitogram represents the number of protein bands in the SDS gel or blot and areas under the peak represents the density of protein bands. Peaks in the densitogram are labeled with an apparent size of the protein, determined using a standard protein ladder protein marker. Color code is used to mark the different proteins in densitogram.

To find the contribution of PSII antenna complex proteins in anti-DMPO blot, the NC membranes were probed with different PSII antenna complex protein antibodies (Figure 6). To detect the protein bands originated from CP43 and CP47 PSII antenna proteins, the blots were probed with anti-CP43 and anti-CP47 antibodies raised against the CP43 and CP47 proteins, respectively. One prominent protein band with an apparent molecular weight of 43 kDa (CP43 protein band) and one prominent protein band with an apparent molecular weight of 68 kDa was detected when the blot was probed with the anti-CP43 antibody. When anti-CP47 antibody was used, a band with an apparent molecular weight of 47 kDa (CP47 protein band) and two weak intensity protein bands with an apparent molecular weight of 52 and 68 kDa above 47 kDa were detected. Probing of the blot with an anti-Lhcb3 antibody raised against Lhcb3 protein showed one protein band with an apparent molecular weight of 23 kDa (Lhcb3 protein band) and one with an apparent molecular weight of 55 kDa. After exposure to high red light, a significant decrease in the protein band densities of CP43 and CP47 proteins was observed. Interestingly, the band density of 23 kDa protein had a small decrease, whereas the band density of 55 kDa protein band significantly increased under high red light. Quantification of protein bands in each lane of blots probed with anti-CP43, anti-CP47 and anti-Lhcb3 antibodies by densitogram showed almost complete loss of CP43 and CP47 protein band and less than half decrease in Lhcb3 protein band density. The decrease in Lhcb3 protein band density was accompanied by several folds increase in the 55 kDa protein band density. Based on the comparison of these blots with anti-DMPO blot of the same samples performed in parallel, it was concluded that protein radicals are formed on the aggregates involving CP43, CP47 and Lhcb3 proteins. These results provide clear evidence that similarly to PSII reaction center proteins, PSII antenna complex proteins are also involved in aggregation and radical formation on the aggregates.

## Discussion

Under the natural environment, plants are exposed to high light associated with the formation of ROS known to cause oxidative damage to biomolecules (Figures 1, 2). Proteins cover approximately 68% of the dry weight of cells and tissues and are therefore potentially the major targets for oxidative damage. Using the immuno-spin trapping technique, we presented here that exposure of Arabidopsis leaf and chloroplasts to high light results in the formation of protein radicals in leaf and chloroplasts (Figures 3A,B). In this study, we provided a characterization of protein radicals in the thylakoid membranes exposed to high light. Our results show the formation of protein radicals on two fragments at approximately 18 and 23 kDa and three aggregates at approximately 41, 55 and 68 kDa (Figure 5). Protein cleavage occurs via β-scission of protein alkoxyl radical known to form carbon-centered radical on the C-terminal and N-terminal fragments. Our results show that the 18 kDa protein band might represent aggregate which arises from two 9 kDa fragments of the C-terminus of D1 protein, two α-subunits of cyt *b*_559_ protein or C-terminus of the D1 and α-subunit of cyt *b*_559_ protein. In agreement with this, it was shown that 18 kDa protein may arise from the C-terminus of the D1 protein (Kale et al., 2017). The 18 kDa protein band might arise from the degradation of D1 protein which may be due to the cleavage in the lumenal loop joining helices C and D (Aro et al., 1990; Shipton and Barber, 1991; Barbato et al., 1992b). The cross-linking of a protein radical with another protein radical results in the formation of protein aggregate. Based on the observation that 41 kDa band was observed when blot was probed with anti-D1 antibody and anti-cyt *b*_559_ α-subunit antibody, it is very likely that protein radical is formed on an aggregate of the D1 protein and the α-subunit of cyt *b*_559_ (Barbato et al., 1992a, 1995; Yamamoto, 2001; Lupínková and Komenda, 2004; Yamamoto et al., 2008). The observation that 55 kDa band appeared when blot was probed with anti-D1, anti- C-terminal, anti-D-*de* loop and anti-Lhcb3 antibodies suggests that protein radical is formed on: (i) the aggregates of 23 kDa fragment of D1 protein and D1 protein, (ii) the aggregates of three 18 kDa fragments of D1 protein, (iii) the aggregates of Lhcb3 protein and C-terminal of D1 protein, (iv) the aggregates of two Lhcb3 proteins. It has been recently reported that light-driven trimer to monomer transition is associated with the appearance of LHCII dimers (Janik et al., 2013, 2017). Similarly, the finding that 68 kDa band was observed when the blot was probed using anti-D1, anti-D2 and anti-CP43 antibodies, reveals that protein radical is formed either on D1/D2 or 23 kDa fragment D1/CP43 protein aggregates as reported in the previous reports (Ishikawa et al., 1999; Henmi et al., 2003). The appearance of 68 kDa band when the blot was probed using an anti-cyt *b*_559_ α-subunit, anti-D-*de* loop and anti-D2 antibodies suggests the formation of an aggregate of these peptides. Our results are in agreement with published literature in past, that under high light illumination the D1 protein cross-links covalently or aggregates non-covalently with the nearby polypeptides in PS II complexes (Aro et al., 1993; Barber, 1998; Komenda et al., 2006; Edelman and Mattoo, 2008; Yamamoto et al., 2008).

## Conclusion

In conclusion, we used the immuno-spin trapping technique to visualize the formation of protein radicals in plant cells by using laser confocal scanning microscopy and characterized the PSII protein oxidized during high light illumination. Formation of protein radicals leads to formation of protein fragments and aggregates and subsequently to formation of protein radicals on these fragments and aggregates. The use of immuno-spin trapping opens new opportunities to study the role of protein radicals in the overall understanding of plant behavior for its survival during the oxidative stress.

## Data Availability

All datasets generated for this study are included in the manuscript and/or the Supplementary Files.

## Author Contributions

PP and AK contributed to the conception and design of the work and drafted the manuscript. AK measured and analyzed the data on immunoblots. AP and MS performed the confocal measurements. AK, AP, and PP performed the data interpretation. All authors approved the final version of the manuscript.

## Conflict of Interest Statement

The authors declare that the research was conducted in the absence of any commercial or financial relationships that could be construed as a potential conflict of interest.

## References

[B1] Aro E. M. Hundal T. Carlberg I. Andersson B. (1990). Invitro studies on light-induced inhibition of photosystem-ii and d1-protein degradation at low-temperatures. *Biochimica Et Biophysica Acta* 1019 269–275. 10.1016/0005-2728(90)90204-h

[B2] Aro E. M. Virgin I. Andersson B. (1993). Photoinhibition of Photosystem Iinactivation, I., protein damage and turnover. *Biochim. Biophys. Acta.* 1143 113–134. 10.1016/0005-2728(93)90134-2 8318516

[B3] Barbato R. Friso G. Ponticos M. Barber J. (1995). Characterization of the light-induced cross-linking of the α-subunit of cytochrome *b*_559_ and the D1 protein in isolated photosystem-ii reaction centers. *J. Biol. Chem.* 270 24032–24037. 10.1074/jbc.270.41.24032 7592601

[B4] Barbato R. Frizzo A. Friso G. Rigoni F. Giacometti G. M. (1992a). Photoinduced degradation of the d1 protein in isolated thylakoids and various photosystem-ii particles after donor-site inactivations - detection of a C-terminal 16 Kda fragment. *FEBS Lett.* 304 136–140. 10.1016/0014-5793(92)80604-f 1618312

[B5] Barbato R. Friso G. Rigoni F. Frizzo A. Giacometti G. M. (1992b). Characterization of a 41 kDa photoinhibition adduct in isolated photosystem II reaction centres. *FEBS Lett.* 309 165–169. 10.1016/0014-5793(92)81087-3 1505680

[B6] Barber J. (1998). Photosystem two. *Biochim. Biophys. Acta.* 1365 269–277. 9693741 10.1016/s0005-2728(98)00079-6

[B7] Brunelle J. L. Green R. (2014). Chapter thirteen - coomassie blue staining. *Methods Enzymol.* 541 161–167. 24674070 10.1016/B978-0-12-420119-4.00013-6

[B8] Casazza A. P. Tarantino D. Soave C. (2001). Preparation and functional characterization of thylakoids from *Arabidopsis thaliana*. *Photosynth. Res.* 68 175–180. 16228340 10.1023/A:1011818021875

[B9] Choudhury F. K. Rivero R. M. Blumwald E. Mittler R. (2017). Reactive oxygen species, abiotic stress and stress combination. *Plant J.* 90 856–867. 10.1111/tpj.13299 27801967

[B10] Davies M. J. (2016). Protein oxidation and peroxidation. *Biochem. J.* 473 805–825. 10.1042/bj20151227 27026395 PMC4819570

[B11] Dietz K. J. Turkan I. Krieger-Liszkay A. (2016). Redox- and reactive oxygen species-dependent signaling into and out of the photosynthesizing chloroplast. *Plant Physiol.* 171 1541–1550. 10.1104/pp.16.00375 27255485 PMC4936569

[B12] Edelman M. Mattoo A. K. (2008). D1-protein dynamics in photosystem II: the lingering enigma. *Photosynth. Res.* 98 609–620. 10.1007/s11120-008-9342-x 18709440

[B13] Fischer B. B. Hideg E. Krieger-Liszkay A. (2013). Production, detection, and signaling of singlet oxygen in photosynthetic organisms. *Anti. Redox Signal* 18 2145–2162. 10.1089/ars.2012.5124 23320833

[B14] Frankel L. K. Sallans L. Limbach P. A. Bricker T. M. (2012). Identification of oxidized amino acid residues in the vicinity of the Mn4CaO5 cluster of photosystem II: implications for the identification of oxygen channels within the photosystem. *Biochemistry* 51 6371–6377. 10.1021/bi300650n 22827410 PMC3448023

[B15] Frankel L. K. Sallans L. Limbach P. A. Bricker T. M. (2013). Oxidized amino acid residues in the vicinity of Q(A) and Pheo(D1) of the photosystem II reaction center: putative generation sites of reducing-side reactive oxygen species. *Plos One* 8:7. 10.1371/journal.pone.0058042 23469138 PMC3585169

[B16] Gomez-Mejiba S. E. Zhai Z. L. Della-Vedova M. C. Munoz M. D. Chatterjee S. Towner R. A. (2014). Immuno-spin trapping from biochemistry to medicine: advances, challenges, and pitfalls. Focus on protein-centered radicals. *Biochim. Biophys. Acta Gen. Subj.* 1840 722–729. 10.1016/j.bbagen.2013.04.039 23644035 PMC4078024

[B17] Henmi T. Yamasaki H. Sakuma S. Tomokawa Y. Tamura N. Shen J. R. (2003). Dynamic interaction between the D1 protein, CP43 and OEC33 at the lumenal side of photosystem II in spinach chloroplasts: evidence from light-induced cross-linking of the proteins in the donor-side photoinhibition. *Plant Cell Physiol.* 44 451–456. 10.1093/pcp/pcg049 12721387

[B18] Ishikawa Y. Nakatani E. Henmi T. Ferjani A. Harada Y. Tamura N. (1999). Turnover of the aggregates and cross-linked products of the D1 protein generated by acceptor-side photoinhibition of photosystem II. *Biochim. Biophys. Acta.* 1413 147–158. 10.1016/s0005-2728(99)00093-6 10556627

[B19] Janik E. Bednarska J. Sowinski K. Luchowski R. Zubik M. Grudzinski W. (2017). Light-induced formation of dimeric LHCII. *Photosynth Res.* 132 265–276. 10.1007/s11120-017-0387-6 28425025 PMC5443882

[B20] Janik E. Bednarska J. Zubik M. Puzio M. Luchowski R. Grudzinski W. (2013). Molecular architecture of plant thylakoids under physiological and light stress conditions: a study of lipid-light-harvesting complex II model membranes. *Plant Cell* 25 2155–2170. 10.1105/tpc.113.113076 23898030 PMC3723618

[B21] Kale R. Hebert A. E. Frankel L. K. Sallans L. Bricker T. M. Pospíšil P. (2017). Amino acid oxidation of the D1 and D2 proteins by oxygen radicals during photoinhibition of Photosystem II. *Proc. Natl. Acad. Sci. U. S. A.* 114 2988–2993. 10.1073/pnas.1618922114 28265052 PMC5358366

[B22] Komenda J. Kuviková S. Lupínková L. Masojídek J. (2006). *Biogenesis and Structural Dynamics of the Photosystem II Complex.* Berlin: Springer.

[B23] Kumar A. Prasad A. Sedlářová M. Pospíšil P. (2018). Data on detection of singlet oxygen, hydroxyl radical and organic radical in Arabidopsis thaliana. *Data Brief* 21 2246–2252. 10.1016/j.dib.2018.11.033 30555863 PMC6276547

[B24] Kumar A. Prasad A. Sedlářová M. Pospíšil P. (2019). Organic radical imaging in plants: focus on protein radicals. *Free Radic. Biol. Med.* 130 568–575. 10.1016/j.freeradbiomed.2018.10.428 30352303

[B25] Laloi C. Havaux M. (2015). Key players of singlet oxygen-induced cell death in plants. *Front. Plant Sci.* 6:39. 10.3389/fpls.2015.00039 25699067 PMC4316694

[B26] Lupínková L. Komenda J. (2004). Oxidative modifications of the photosystem II D1 protein by reactive oxygen species: from isolated protein to cyanobacterial cells. *Photochem. Photobiol.* 79 152–162. 10.1111/j.1751-1097.2004.tb00005.x 15068028

[B27] Mason R. P. (2016). Imaging free radicals in organelles, cells, tissue, and in vivo with immuno-spin trapping. *Redox. Biol.* 8 422–429. 10.1016/j.redox.2016.04.003 27203617 PMC4878322

[B28] Mattila H. Khorobrykh S. Havurinne V. Tyystjarvi E. (2015). Reactive oxygen species: reactions and detection from photosynthetic tissues. *J. photochem. Photobiol. B Biol.* 152(Pt B), 176–214. 10.1016/j.jphotobiol.2015.10.001 26498710

[B29] Mittler R. (2017). ROS are good. *Trends Plant Sci.* 22 11–19. 10.1016/j.tplants.2016.08.002 27666517

[B30] Moan J. Wold E. (1979). Detection of singlet oxygen production by ESR. *Nature* 279 450–451. 10.1038/279450a0 16068192

[B31] Munoz M. D. Gutierrez L. J. Delignat S. Russick J. Mejiba S. E. G. Lacroix-Desmazes S. (2019). The nitrone spin trap 5,5-dimethyl-1-pyrroline N -oxide binds to toll -like receptor-2-TIR-BB-loop domain and dampens downstream inflammatory signaling. *Biochimica Et Biophysica Acta Molecular Basis of Disease* 1865 1152–1159. 10.1016/j.bbadis.2019.01.005 30684639

[B32] Murata N. Takahashi S. Nishiyama Y. Allakhverdiev S. I. (2007). Photoinhibition of photosystem II under environmental stress. *Biochim. Biophys. Acta.* 1767 414–421. 10.1016/j.bbabio.2006.11.019 17207454

[B33] Pospíšil P. (2012). Molecular mechanisms of production and scavenging of reactive oxygen species by photosystem II. *Biochim. Biophys. Acta.* 1817 218–231. 10.1016/j.bbabio.2011.05.017 21641332

[B34] Pospíšil P. (2014). The role of metals in production and scavenging of reactive oxygen species in photosystem II. *Plant Cell Physiol.* 55 1224–1232. 10.1093/pcp/pcu053 24771559

[B35] Pou S. Ramos C. L. Gladwell T. Renks E. Centra M. Young D. (1994). A kinetic approach to the selection of a sensitive spin trapping system for the detection of hydroxyl radical. *Anal. Biochem.* 217 76–83. 10.1006/abio.1994.1085 8203741

[B36] Prasad A. Sedlářová M. Pospíšil P. (2018). Singlet oxygen imaging using fluorescent probe singlet oxygen sensor green in photosynthetic organisms. *Sci. Rep.* 8:13685. 10.1038/s41598-018-31638-5 30209276 PMC6135792

[B37] Schagger H. (2006). Tricine-SDS-PAGE. *Nat. Protoc.* 1 16–22. 10.1038/nprot.2006.4 17406207

[B38] Schmitt F. J. Renger G. Friedrich T. Kreslavski V. D. Zharmukhamedov S. K. Los D. A. (2014). Reactive oxygen species: re-evaluation of generation, monitoring and role in stress-signaling in phototrophic organisms. *Biochim. Biophys. Acta.* 1837 835–848. 10.1016/j.bbabio.2014.02.005 24530357

[B39] Seigneurin-Berny D. Salvi D. Joyard J. Rolland N. (2008). Purification of intact chloroplasts from Arabidopsis and spinach leaves by isopycnic centrifugation. *Curr. Protocol. Cell Biol.* 40 3.30.1–3.30.14. 10.1002/0471143030.cb0330s40 18819091

[B40] Shipton C. A. Barber J. (1991). Photoinduced degradation of the D1 polypeptide in isolated reaction centers of photosystem II: evidence for an autoproteolytic process triggered by the oxidizing side of the photosystem. *Proc. Natl. Acad. Sci. U. S. A.* 88 6691–6695. 10.1073/pnas.88.15.6691 1862094 PMC52154

[B41] Telfer A. (2014). Singlet oxygen production by PSII under light stress: mechanism, detection and the protective role of beta-carotene. *Plant Cell Physiol.* 55 1216–1223. 10.1093/pcp/pcu040 24566536 PMC4080269

[B42] Weisz D. A. Gross M. L. Pakrasi H. B. (2017). Reactive oxygen species leave a damage trail that reveals water channels in photosystem II. *Sci. Adv.* 3:eaao3013. 10.1126/sciadv.aao3013 29159285 PMC5693562

[B43] Yamamoto Y. (2001). Quality control of photosystem II. *Plant Cell Physiol.* 42 121–128. 10.1093/pcp/pce022 11230565

[B44] Yamamoto Y. Aminaka R. Yoshioka M. Khatoon M. Komayama K. Takenaka D. (2008). Quality control of photosystem II: impact of light and heat stresses. *Photosynth Res.* 98 589–608. 10.1007/s11120-008-9372-4 18937045

