# Supplementary material for: Characterization of Protein Radicals in Arabidopsis
Source: Front Physiol. 2019 Aug 13;10:958. doi: 10.3389/fphys.2019.00958 (PMC6700370; doi:10.3389/fphys.2019.00958)
Supplement: Supplementary file 1 [file Data_Sheet_1.pdf]

Characterization of protein radicals in Arabidopsis

Aditya Kumar[1], Ankush Prasad[1], Michaela Sedlářová[2], Pavel Pospíšil[1*]

[1]Department of Biophysics, Centre of the Region Haná for Biotechnological and Agricultural Research, Faculty of Science, Palacký University, Olomouc, Czech Republic, [2]Department of Botany, Faculty of Science, Palacký University, Šlechtitelů 27, 783 71 Olomouc, Czech Republic,

[2]Department of Botany, Faculty of Science, Palacký University, Šlechtitelů 27, 783 71 Olomouc, Czech Republic

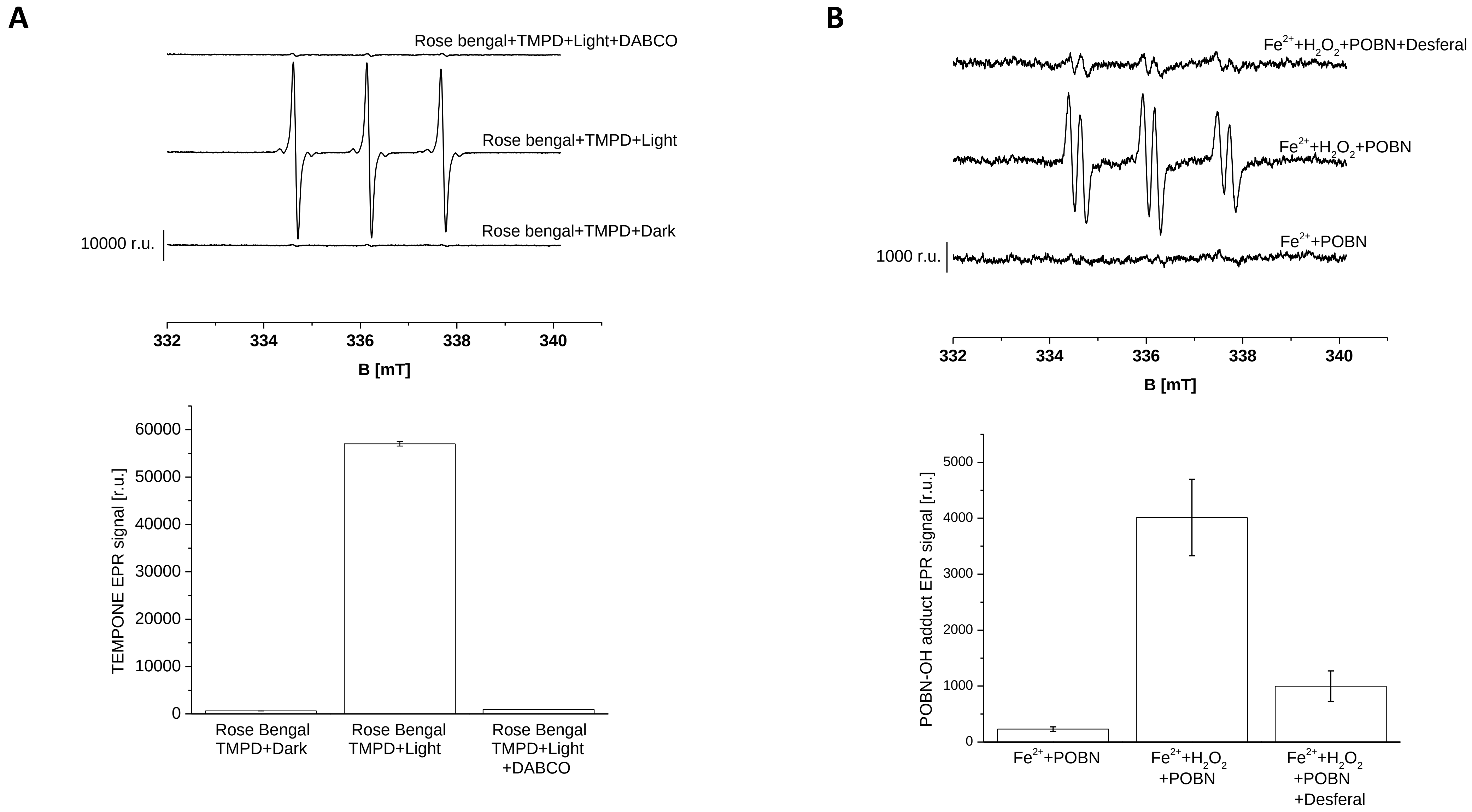

Figure S1. Detection of singlet oxygen (A) and hydroxyl radical (B) in chemical system. Detection of singlet oxygen ($^1O_2$) and hydroxyl radical ($HO^{\bullet}$) by EPR spin trapping spectroscopy. **(A)** Rose bengal-induced TEMPONE EPR spectra: Rose bengal (50 μM) with 50 mM TMPD in dark and under white light illumination (1500 μmol photons m^{-2} s^{-1}, 1 min) in the absence and presence of 25 mM DABCO. **(B)** Fenton's reagent-induced POBN (4-pyridyl-1-oxide-N-tert-butylnitrone)-OH adduct EPR spectra: 5 mM $FeSO_4$ with 50 mM POBN in the absence (control) and presence of 1 mM H_2O_2. POBN-OH adduct EPR spectra was also measured in the presence of desferal (5mM). The lower panels shows the mean and standard deviation of EPR signal intensity (where, n=3).

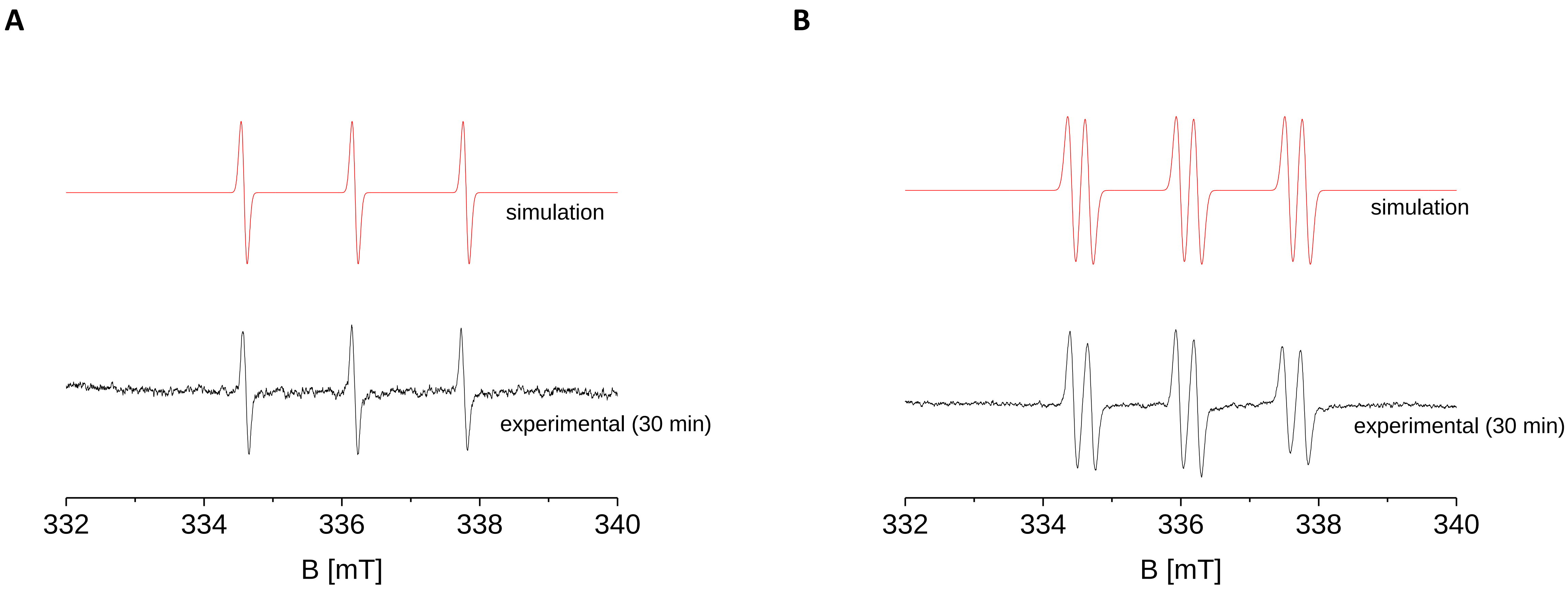

Figure S2. Simulation of EPR spectra **A.** Thylakoid membranes (50 µg Chl ml^{-1}) in buffer containing 50 mM TMPD illuminated with high white light (1500 µmol photons m^{-2} s^{-1}, 30 min) and simulated TEMPONE EPR spectrum obtained using the hyperfine coupling constant a^N = 16.10 G[1]. B. Thylakoid membranes (100 µg Chl ml^{-1}) in 50 mM POBN containing 170 mM ethanol with high white light (1500 µmol photons m^{-2} s^{-1}, 30 min) and simulated POBN-CH(CH_3)OH adduct EPR spectrum obtained using the hyperfine coupling constants a^N = 15.75 G and a^H = 2.40 G[2].

Reference:

1. J. Moan, E. Wold, Detection of singlet oxygen production by ESR, Nature 279, 450–451 (1979).
2. S. Pou, C.L. Ramos, T. Gladwell, E. Renks, M. Centra, D. Young, M.S. Cohen, G.M. Rosen, A kinetic approach to the selection of a sensitive spin trapping system for the detection of hydroxyl radical, Anal Biochem 217(1) (1994) 76-83.
