# Supplementary material for: Characterization of Protein Radicals in Arabidopsis
Source: Front Physiol. 2019 Aug 13;10:958. doi: 10.3389/fphys.2019.00958 (PMC6700370; doi:10.3389/fphys.2019.00958)
Supplement: Supplementary file 2 [file Data_Sheet_2.pdf]

abcam

Product datasheet

Anti-DMPO Nitrone Adduct antibody [N1664A] ab104902

1 References 2 Images

Overview

**Product name**	Anti-DMPO Nitrone Adduct antibody [N1664A]
**Description**	Mouse monoclonal [N1664A] to DMPO Nitrone Adduct
**Host species**	Mouse
**Specificity**	Detects ~90kDa. Recognizes DMPO, DMPO-octanoic acid, DMPO-protein adducts and DMPO-DNA adducts. Does not cross-react with non-adducted proteins or DNA.
**Tested applications**	**Suitable for:** WB, ELISA, ICC/IF, IP
**Species reactivity**	**Reacts with:** Species independent
**Immunogen**	5,5-dimethyl-2-(8-octanoic acid)-1-pyrrolone-N-oxide conjugated to Ovalbumin.

Properties

**Form**	Liquid
**Storage instructions**	Shipped at 4°C. Upon delivery aliquot and store at -20°C. Avoid freeze / thaw cycles.
**Storage buffer**	Preservative: 0.05% Sodium Azide<br>Constituents: 50% Glycerol, Tris buffered saline, pH 7.4
**Purity**	Protein G purified
**Clonality**	Monoclonal
**Clone number**	N1664A
**Isotype**	IgG1

Applications

Our Abpromise guarantee covers the use of **ab104902** in the following tested applications.

The application notes include recommended starting dilutions; optimal dilutions/concentrations should be determined by the end user.

Application	Abreviews	Notes
WB		Use a concentration of 1 - 10 µg/ml.

Application	Abreviews	Notes
ELISA		Use a concentration of 1 - 10 µg/ml.
ICC/IF		Use a concentration of 1 - 10 µg/ml.
IP		Use at an assay dependent dilution. Follow the method described by Chatterjee, S. *et. al.* PubMed 19049863.

Target

Relevance For measurement of protein radicals; some proteins with endogenous peroxidase activity are especially susceptible to forming DMPO adducts.

Images

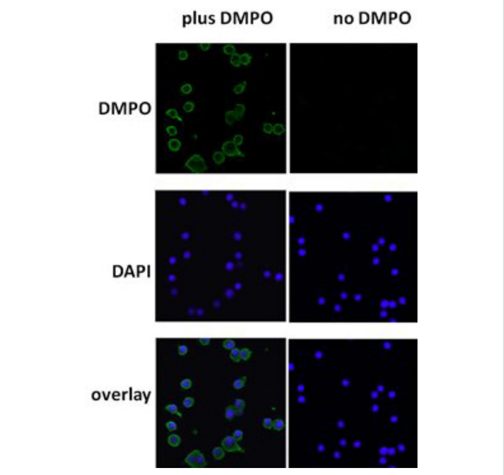

Immunocytochemistry/ Immunofluorescence - Anti-DMPO Nitrone Adduct antibody [N1664A] (ab104902)

Immuncytochemistry/Immunofluorescence analysis of Mouse macrophage cells labelling DMPO Nitrone Adduct with ab104902 at 10µg/mL. Left - with DMPO, Right - no DMPO. Top to bottom - DMPO, DAPI, overlay.

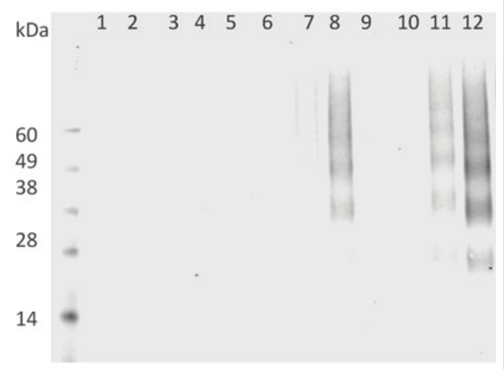

Western blot - Anti-DMPO Nitrone Adduct antibody [N1664A] (ab104902)

All lanes : Anti-DMPO Nitrone Adduct antibody [N1664A] (ab104902) at 10 µg/ml

Lane 1 : 10µM Hb
Lane 2 : 10µM Hb + 100µM HOCL
Lane 3 : 10µM Hb + 500µM HOCL
Lane 4 : 10µM Hb + 1000µM HOCL
Lane 5 : 10µM Hb with 20mM DMPO
Lane 6 : 10µM Hb + 100µM HOCL with 20mM DMPO
Lane 7 : 10µM Hb + 500µM HOCL with 20mM DMPO
Lane 8 : 10µM Hb + 1000µM HOCL with 20mM DMPO
Lane 9 : 10µM Hb with 100mM DMPO

Lane 10 : 10µM Hb + 100µM HOCL with 100mM DMPO

Lane 11 : 10µM Hb + 500µM HOCL with 100mM DMPO

Lane 12 : 10µM Hb + 1000µM HOCL with 100mM DMPO

Please note: All products are "FOR RESEARCH USE ONLY AND ARE NOT INTENDED FOR DIAGNOSTIC OR THERAPEUTIC USE"

Our Abpromise to you: Quality guaranteed and expert technical support

- Replacement or refund for products not performing as stated on the datasheet
- Valid for 12 months from date of delivery
- Response to your inquiry within 24 hours

- We provide support in Chinese, English, French, German, Japanese and Spanish
- Extensive multi-media technical resources to help you
- We investigate all quality concerns to ensure our products perform to the highest standards

If the product does not perform as described on this datasheet, we will offer a refund or replacement. For full details of the Abpromise, please visit https://www.abcam.com/abpromise or contact our technical team.

Terms and conditions

- Guarantee only valid for products bought direct from Abcam or one of our authorized distributors
